# Supplementary material for: Hybrid Models and Biological Model Reduction with PyDSTool
Source: PLoS Comput Biol. 2012 Aug 9;8(8):e1002628. doi: 10.1371/journal.pcbi.1002628 (PMC3415397; doi:10.1371/journal.pcbi.1002628)
Supplement: Text S4 — Complete source code for the PyDSTool package (version 0.88.120504). Includes API documentation and help files linking to web pages. This file is identical to the current public release on Sourceforge.net. (ZIP) [file pcbi.1002628.s004.zip › PyDSTool/html/PyDSTool.Toolbox.optimizers.line_search-module.html]

xml version="1.0" encoding="ascii"?


PyDSTool.Toolbox.optimizers.line\_search


| Home | Trees | Indices | Help | PyDSTool |
| --- | --- | --- | --- | --- |

Package PyDSTool ::
Package Toolbox ::
Package optimizers ::
Package line\_search

[hide private]

[frames] | no frames]

# Package line\_search

source code

Module containing the line searchers

Line Searches :

- SimpleLineSearch
  - takes a simple step
- HyperbolicLineSearch
  - takes a step of length 1/(1+iterations)
- DampedLineSearch
  - searches for a candidate by dividing the step by 2 each time
- BacktrackingSearch
  - finds a candidate according to the Armijo rule
- WolfePowellRule
  - finds a candidate according to the standard Wolfe-Powell rules
- StrongWolfePowellRule
  - finds a candidate according to the strong Wolfe-Powell rules
- GoldsteinRule
  - finds a candidate according to the Goldstein rules
- GoldenSectionSearch
  - uses the golden section method for exact line search
- FibonacciSectionSearch
  - uses the Fibonacci section method for exact line search
- QuadraticInterpolationSearch
  - uses the quadratic interpolation method with computation of the
    gradient at the origin for exact line search
- AdaptiveLastStepModifier
  - modifies the last step length depending on the last direction and
    gradient and current gradient and direction
- FixedLastStepModifier
  - modified the last step length with a fixed factor


|  |  |
| --- | --- |
| Submodules | [hide private] |

- **PyDSTool.Toolbox.optimizers.line\_search.adaptive\_last\_step\_modifier**: *Line search decorator that overrides the default alpha\_step value
  with a factor times the last alpha\_step, the factor being a
  function of the current direction and the last direction*
- **PyDSTool.Toolbox.optimizers.line\_search.backtracking\_search**
- **PyDSTool.Toolbox.optimizers.line\_search.barzilai\_borwein\_non\_monotone\_search**
- **PyDSTool.Toolbox.optimizers.line\_search.barzilai\_borwein\_search**
- **PyDSTool.Toolbox.optimizers.line\_search.cubic\_interpolation**: *Line Search with the cubic interpolation method with the
  computation of the gradient of the function*
- **PyDSTool.Toolbox.optimizers.line\_search.damped\_line\_search**: *A damped line search*
- **PyDSTool.Toolbox.optimizers.line\_search.fibonacci\_section**: *Line Search with the Fibonacci section method*
- **PyDSTool.Toolbox.optimizers.line\_search.fixed\_last\_step\_modifier**: *Line search decorator that overrides the default alpha\_step value
  with a factor times the last alpha\_step*
- **PyDSTool.Toolbox.optimizers.line\_search.golden\_section**: *Line Search with the golden section method*
- **PyDSTool.Toolbox.optimizers.line\_search.goldstein\_rule**
- **PyDSTool.Toolbox.optimizers.line\_search.hyperbolic\_line\_search**: *An hyperbolic line search, in fact no searches at all*
- **PyDSTool.Toolbox.optimizers.line\_search.quadratic\_interpolation**: *Line Search with the quadratic interpolation method with the
  computation of the gradient of the function*
- **PyDSTool.Toolbox.optimizers.line\_search.scaled\_line\_search**
- **PyDSTool.Toolbox.optimizers.line\_search.simple\_line\_search**: *A simple line search, in fact no searches at all*
- **PyDSTool.Toolbox.optimizers.line\_search.strong\_wolfe\_powell\_rule**
- **PyDSTool.Toolbox.optimizers.line\_search.wolfe\_powell\_rule**

  


|  |  |
| --- | --- |
| Classes | [hide private] |

SimpleLineSearch  
A simple line search, takes a point, adds a step and returns it

HyperbolicLineSearch  
An inverse line search, takes a point, adds a step
(1/(1+iterations)) and returns it

DampedLineSearch  
A damped line search, takes a point and a direction.

BacktrackingSearch  
The backtracking algorithm for enforcing Armijo rule

WolfePowellRule  
The standard Wolfe-Powell rule for a inexact line search

StrongWolfePowellRule  
The strong Wolfe-Powell rule for a inexact line search

GoldsteinRule  
The Goldstein rule for a inexact line search

GoldenSectionSearch  
Line Search with the golden section method

FibonacciSectionSearch  
Line Search with the Fibonacci section method, optimal section
method

QuadraticInterpolationSearch  
Line Search with the quadratic interpolation when the gradient of
the function is provided

CubicInterpolationSearch  
Line Search with the cubic interpolation when the gradient of the
function is provided

AdaptiveLastStepModifier  
Overrides the default step size and replaces it with a factor times
the last one

FixedLastStepModifier  
Overrides the default step size and replaces it with a factor times
the last one

ScaledLineSearch  
A simple line search, takes a point, adds a step and returns it
Scales step according to given scales of the parameters and ignores
\*magnitude\* of gradient.


|  |  |
| --- | --- |
| Variables | [hide private] |

line\_search\_\_all\_\_ = `['SimpleLineSearch', 'HyperbolicLineSearc...`


|  |  |
| --- | --- |
| Variables Details | [hide private] |

### line\_search\_\_all\_\_

Value:
:   ```
    ['SimpleLineSearch',
     'HyperbolicLineSearch',
     'DampedLineSearch',
     'BacktrackingSearch',
     'WolfePowellRule',
     'StrongWolfePowellRule',
     'GoldsteinRule',
     'GoldenSectionSearch',
    ...
    ```

  


| Home | Trees | Indices | Help | PyDSTool |
| --- | --- | --- | --- | --- |

|  |  |
| --- | --- |
| Generated by Epydoc 3.0.1 on Fri May 4 15:24:05 2012 | http://epydoc.sourceforge.net |
